# Neonatal intestinal diffuse ganglioneuromatosis with plexiform neurofibromas; Diagnostic and management pitfalls—A case report

**DOI:** 10.1002/ccr3.5173

**Published:** 2022-02-13

**Authors:** Madani Essa

**Affiliations:** ^1^ Departments Pediatric Surgery Jazan Faculty of Medicine Jazan University Jazan Saudi Arabia

**Keywords:** intestinal ganglioneuromatosis, intestinal neurofibromatosis, neonatal intestinal obstruction

## Abstract

Neonatal intestinal ganglioneuroma and neurofibromatosis are rare in neonates. It is a challenging pathology to diagnose and treat. A 3‐week‐old full‐term newborn with a birth weight of 3.5 kg of Arabic ethnicity presented to the emergency department with a recurrent refractory intestinal obstruction. The patient has been diagnosed initially with intestinal obstruction secondary to malrotation and then operated again because of adhesion and internal herniation. Finally, after the third laparotomy, the patient was diagnosed with intestinal ganglioneuromatosis and neurofibromatosis. We reviewed the literature on the diagnosis and management of this rare pathology among neonates and to increase the awareness of it. Intestinal ganglioneuromatosis is a rare neonatal pathology. The clinical presentation of intestinal ganglioneuromatosis is similar to that of intestinal obstructions caused by many other diseases. Ganglioneuromatosis is differentiated from other differential diagnoses based on histopathology. Early diagnosis is vital to ensure appropriate management.

## Graphical abstract

Intestinal ganglioneuromatosis presentation is like that of intestinal obstructions caused by many other diseases. Ganglioneuromatosis is differentiated from other differential diagnoses based on histopathology. Multidisciplinary approach for diagnosis and management is needed to avoid unnecessary surgical intervention.

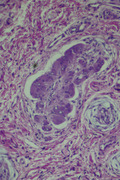

## 1 INTRODUCTION

A ganglioneuroma is a benign neurogenic tumor often diagnosed in children. Although ganglioneuromas typically develop from sympathetic ganglia and adrenal glands, some arise from the viscera. Intestinal ganglioneuromas are rare and more commonly found in children than in adults. Ganglioneuromas consist of three subgroups: (1) polypoid ganglioneuromas, (2) ganglioneuromatous polyposis, and (3) diffuse ganglioneuromatosis.^1^, ^2^ Diffuse intestinal ganglioneuromatosis often leads to thickening of the bowel wall and stricture formation, abdominal pain, and diarrhea.^3^ Diffuse ganglioneuromatosis is typically associated with several diseases, including neurofibromatosis‐1, Cowden syndrome, and multiple endocrine neoplasia type 2B.^3^ In this report, we describe a case of diffuse intestinal ganglioneuromatosis with plexiform neurofibromas in a 3‐week‐old female neonate.

## 2 CASE PRESENTATION

**FIGURE 1:**
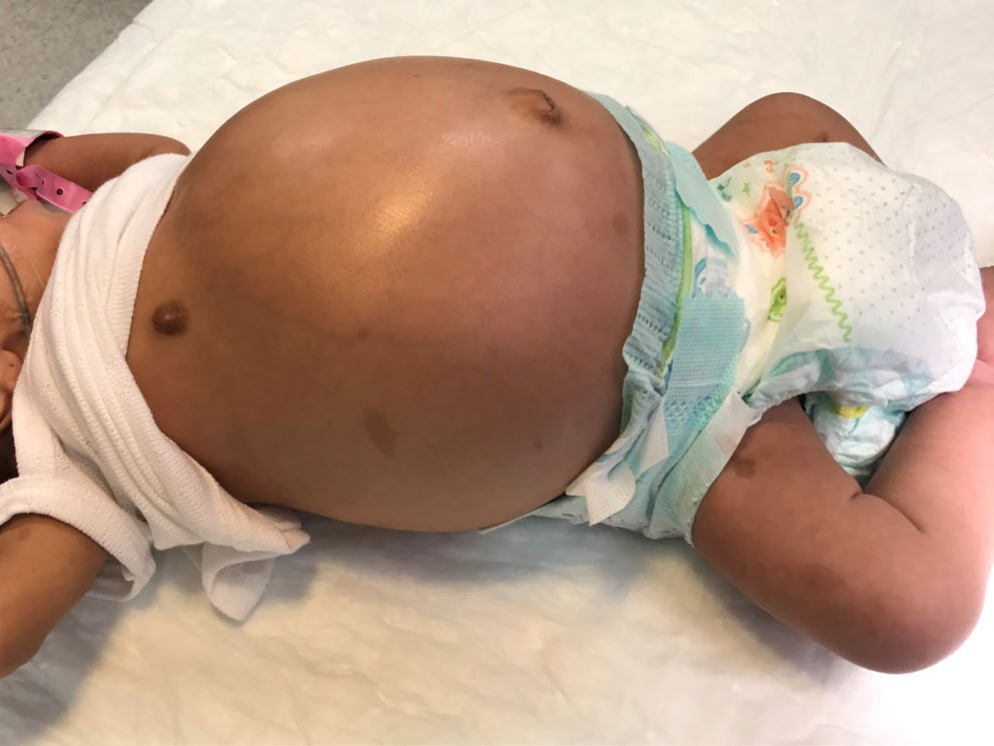
1st presentation to outpatient clinic; abdominal distention, cafe‐au‐lait spots

**FIGURE 2:**
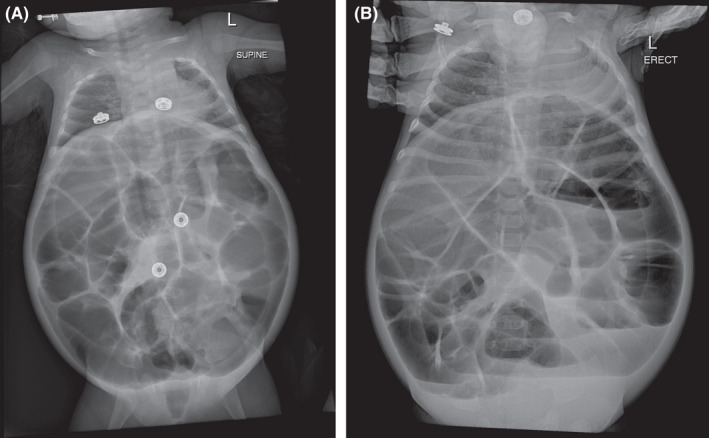
(A) Supine X‐ray, Dilated loops. (B) X‐Ray erect, Air fluid level

**FIGURE 3:**
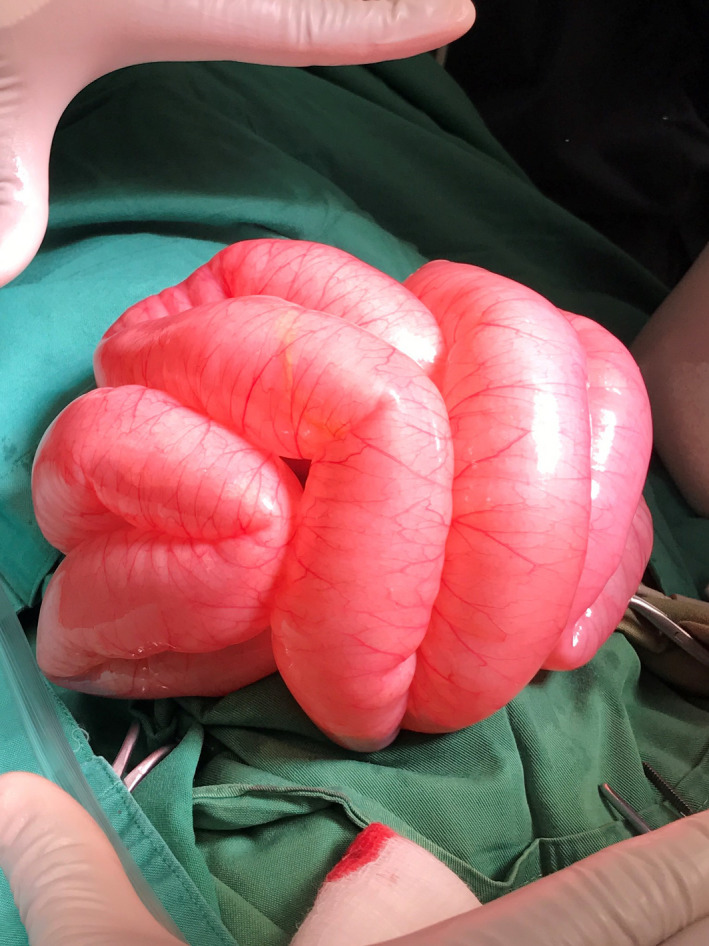
1st lapartomy, dilated intestine with volvulus

A 3‐week‐old female neonate presented to our outpatient clinic with a complaint of abdominal distention with no vomiting or constipation. Small amounts of well‐formed stools were passed on a regular basis. On physical examination; a 3.5 kg female patient with a severely distended abdomen, soft, and lax, with no tenderness. All other gastrointestinal and other systemic findings were relatively normal apart from multiple cafe‐au‐lait spots all over the body (Figure 1). In the laboratory workup, all values were normal. The patient has been scheduled for outpatient follow‐up. Five days later, the patient returned to our emergency department with the complaint of severe abdominal distension, absolute constipation, and vomiting. X‐ray findings indicated severely dilated loops, with multiple air‐fluid levels (Figure 2A, B). An emergency laparotomy was performed. Intraoperatively, the ileum was severely dilated, with volvulus. De‐rotation was done, and the contents were easily evacuated to the colon and passed through the Anus which was mainly gases. The initial diagnosis was malrotation, and an appendectomy was performed electively as apert of malrotation management. Ladd's bands were absent, and the volvulus was mainly due to intestinal loop dilation. Postoperatively, the condition of the patient was fair (Figure 3).

**FIGURE 4:**
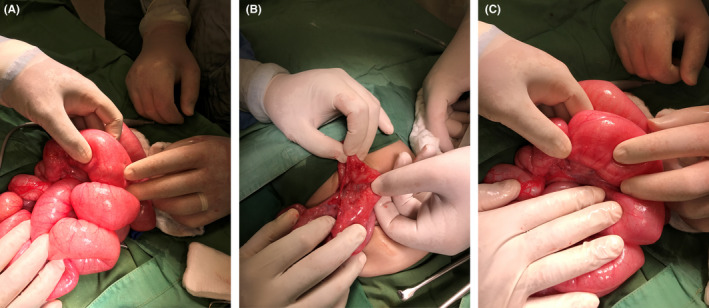
(A) 2nd laparotomy, internal herniation. (B) Adhesion between appendix stump and terminal ileum. (C) Adhesion between appendix stump and terminal ileum

Seven days later, the patient developed severe abdominal distention again, with signs of an intestinal obstruction. A second emergency exploration laparotomy was done. Intraoperatively, we found an adhesion between the stump of the appendix and ileum, with intestinal internal herniation in between. (Figure 4A,B,C). Multiple intestinal biopsies were taken from different sites to rule out Hirschsprung disease; ascending colon, recto‐sigmoid, and stoma site, which was 15 cm proximal to ileocecal. A de‐functioning ileostomy was performed. According to the histopathological results, the ascending colon biopsy specimens revealed nearly normal colonic mucosa, with a slight thickening of the muscularis propria. No specific pathology was detected. The recto‐sigmoid colon biopsy specimens revealed similar changes as seen in the ascending colon biopsy. The biopsy samples from the stoma site of the small intestine revealed atrophy of the mucosal lining, with focal areas of granulation tissue replacing the mucosa. No specific pathological diagnosis was observed. The postoperative recovery of the patient was good, with the disappearance of the distention and a well‐functioning stoma. The patient was discharged 5 days later and scheduled for outpatient clinic follow‐up.

**FIGURE 5:**
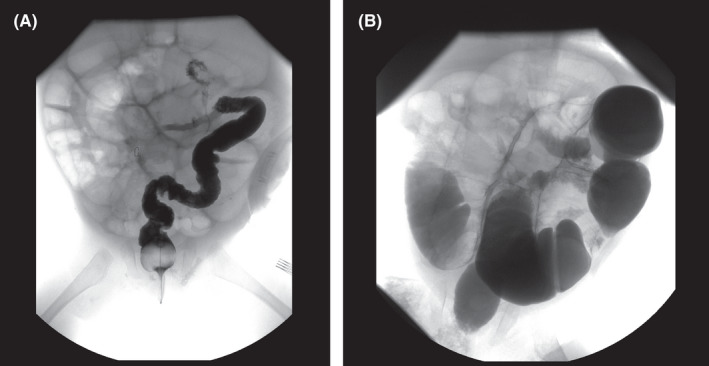
(A) Contrast study; Rectal enema. (B) Contrast study; colostogram

The infant, who lived in a rural area far from the hospital, did not attend the hospital again until 3 months later. The patient presented again with the complaint of an intestinal obstruction. The infant's mother declared that the infant had regular stool motions until 5 days prior to the presentation. On examination, the infant appeared unwell, with severe dehydration and severe abdominal distention indicative of recurrent intestinal obstruction. A diagnostic contrast enema was administered through the anus and ileostomy showed a small bowel obstruction (Figure 5A,B). The initial management involved stabilization in the intensive care unit. The obstruction was initially managed by deflation, with a tube passed through the stoma, which was narrow. The destination relieved a little bit.

**FIGURE 6:**
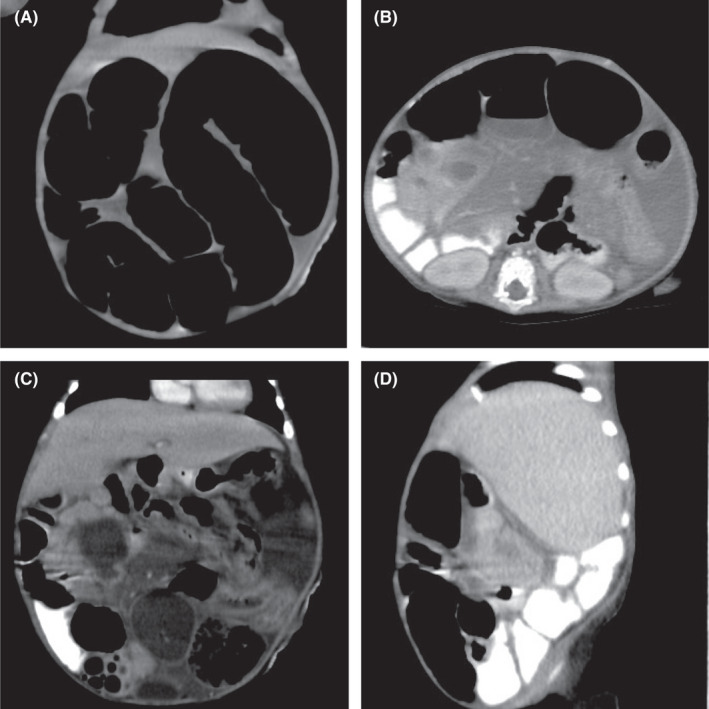
(A) CT; Dilated Bowel loops. (B) CT; thickened bowel wall and mesentery. (C) CT; thickened bowel wall and mesentery. (D) CT; thickened bowel wall and mesentery

**FIGURE 7:**
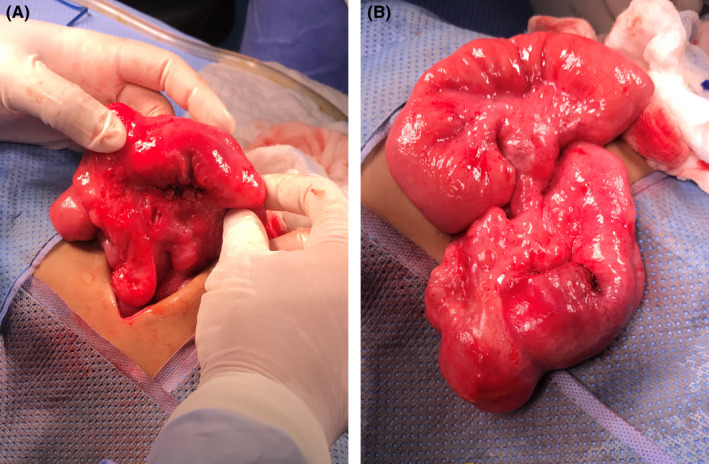
(A) 3rd laparotomy; mesenteric Lymph nods. (B) 3rd laparotomy; cecal mass

A computed tomography (CT) scan performed later showed a transition zone about 4 cm distal to the stoma, a 3‐cm‐long segment of stricture, and a 4‐mm thickened wall, with no evidence of a bowel mass. There was evidence of malrotation of the bowel loops, with reversal of the superior mesenteric artery and superior mesenteric vein, with an abnormal location of the bowel loops (Figure 6A‐D). After a discussion with the infant's family, the patient underwent surgery for the third time. Intraoperatively, a thickened mesentery, with a hard mass at the ileocecal junction with multiple enlarged mesenteric lymph nodes, was observed (Figure 7). The mass was resected, and a specimen was sent to the histopathology department.

Postoperatively, the patient was stable and was discharged in quite a good condition.

**FIGURE 8:**
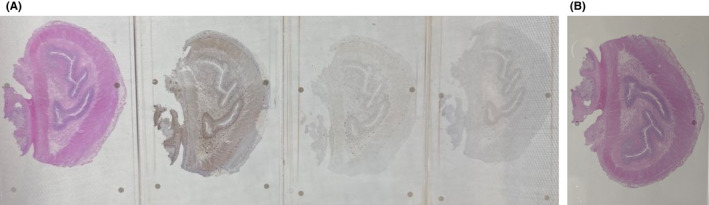
(A) Photograph of the specimen on different slides. The specimen appears reactive to immunohistochemistry. It shows double lumen and a thickened wall in the dilated terminal ileum. (B) Double lumen and a thickened wall in the dilated terminal ileum

**FIGURE 9:**
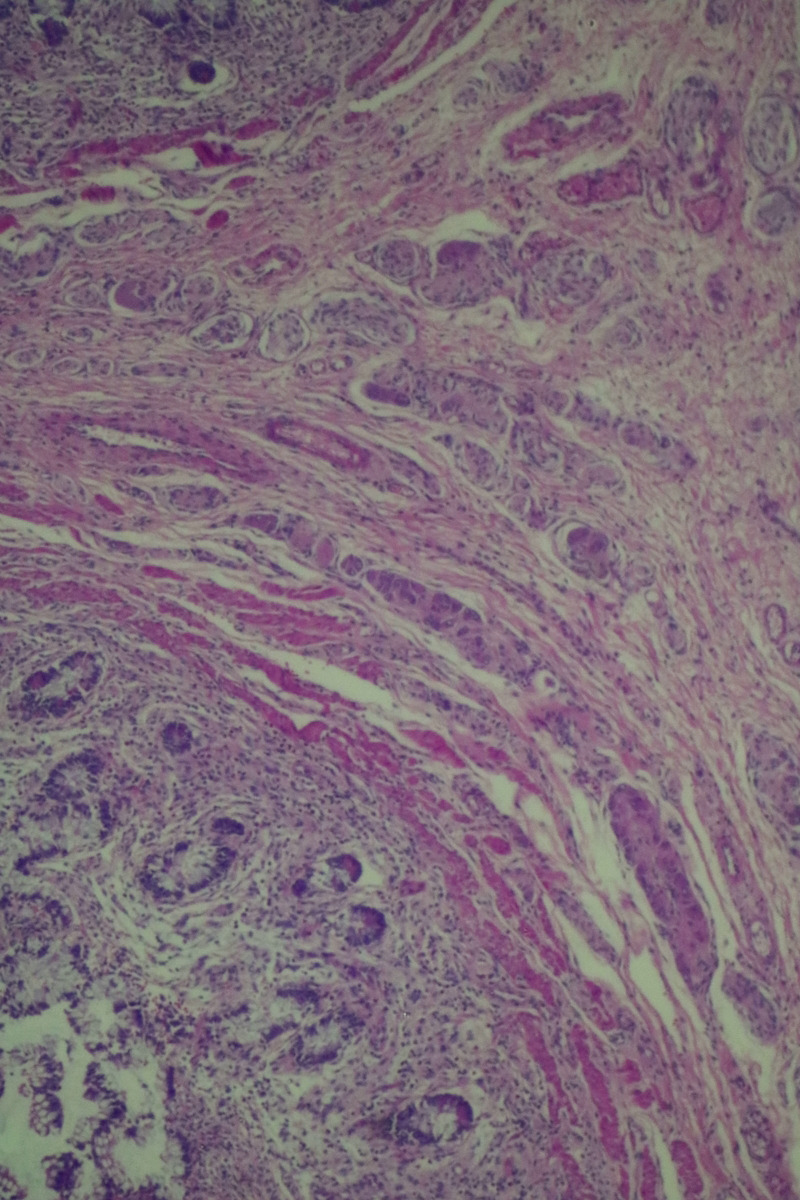
The lamina propria were involved with a tortuous expansion of multiple variable‐sized nerve fascicles (a plexiform neurofibroma; 4× Low power (scanning) objective lens)

**FIGURE 10:**
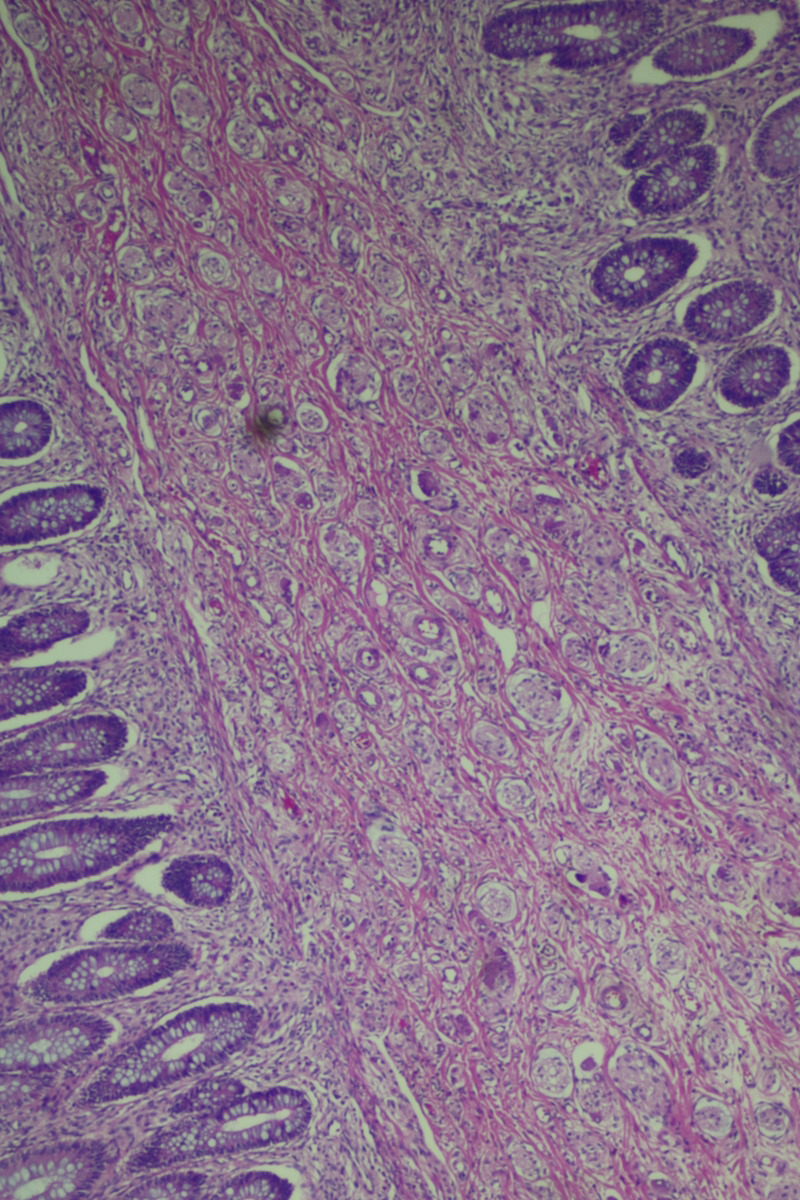
The lamina propria were involved with a tortuous expansion of multiple variable‐sized nerve fascicles (a plexiform neurofibroma; 4× Low power (scanning) objective lens)

**FIGURE 11:**
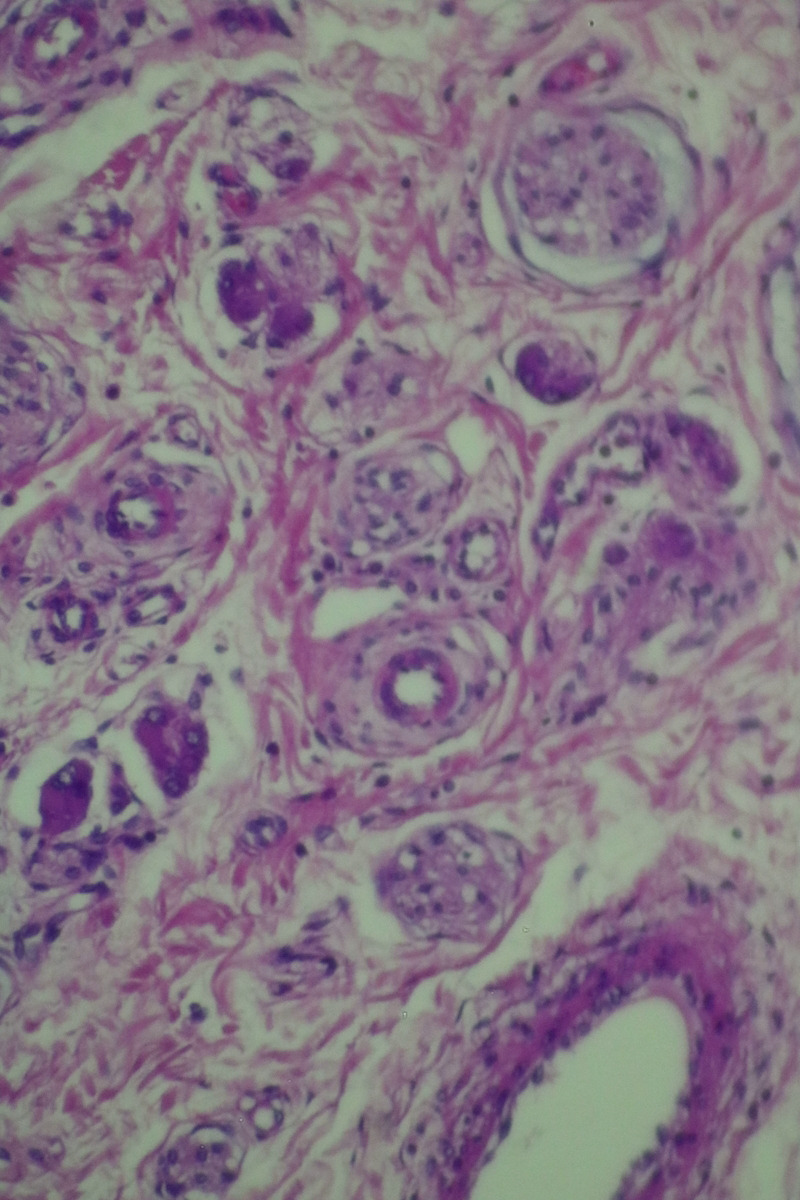
Ganglion proliferation in the lamina propria. Medium power objective (10×)

**FIGURE 12:**
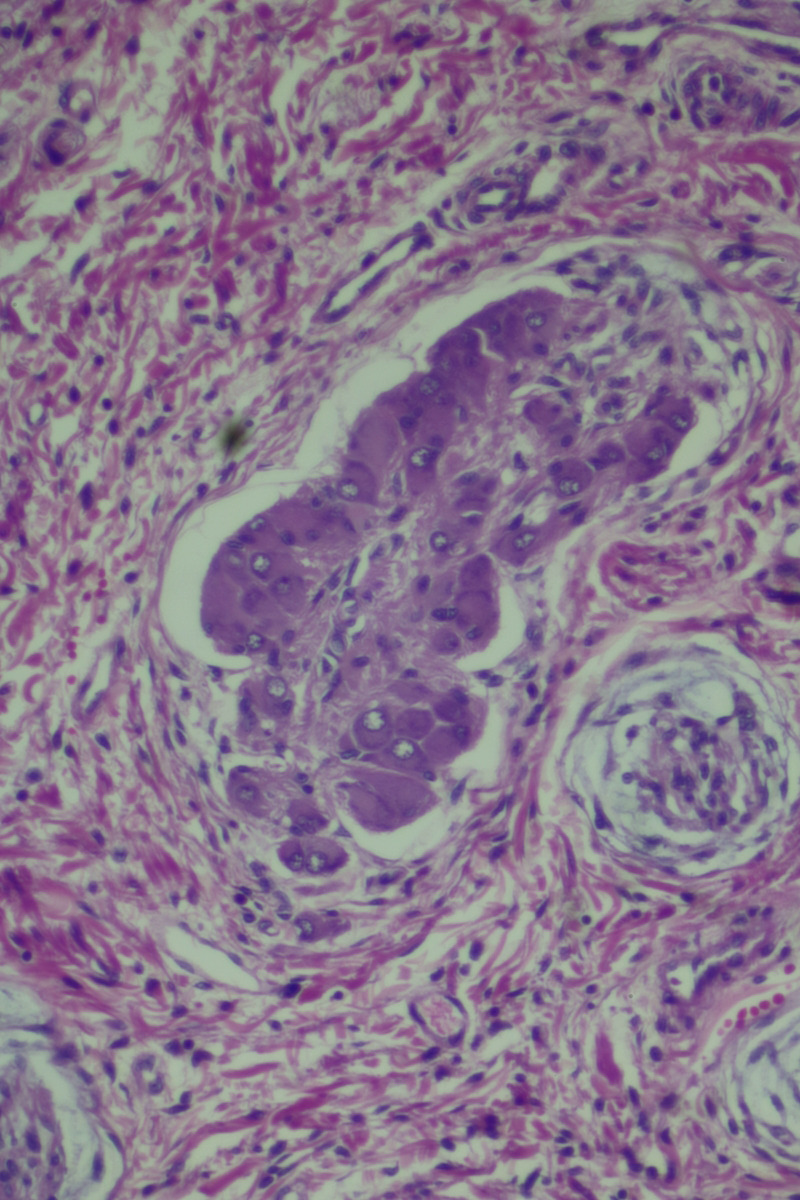
Proliferation of spindle cells, admixed with scattered chronic inflammatory cells throughout the lamina propria. Medium power objective (10×)

**FIGURE 13:**
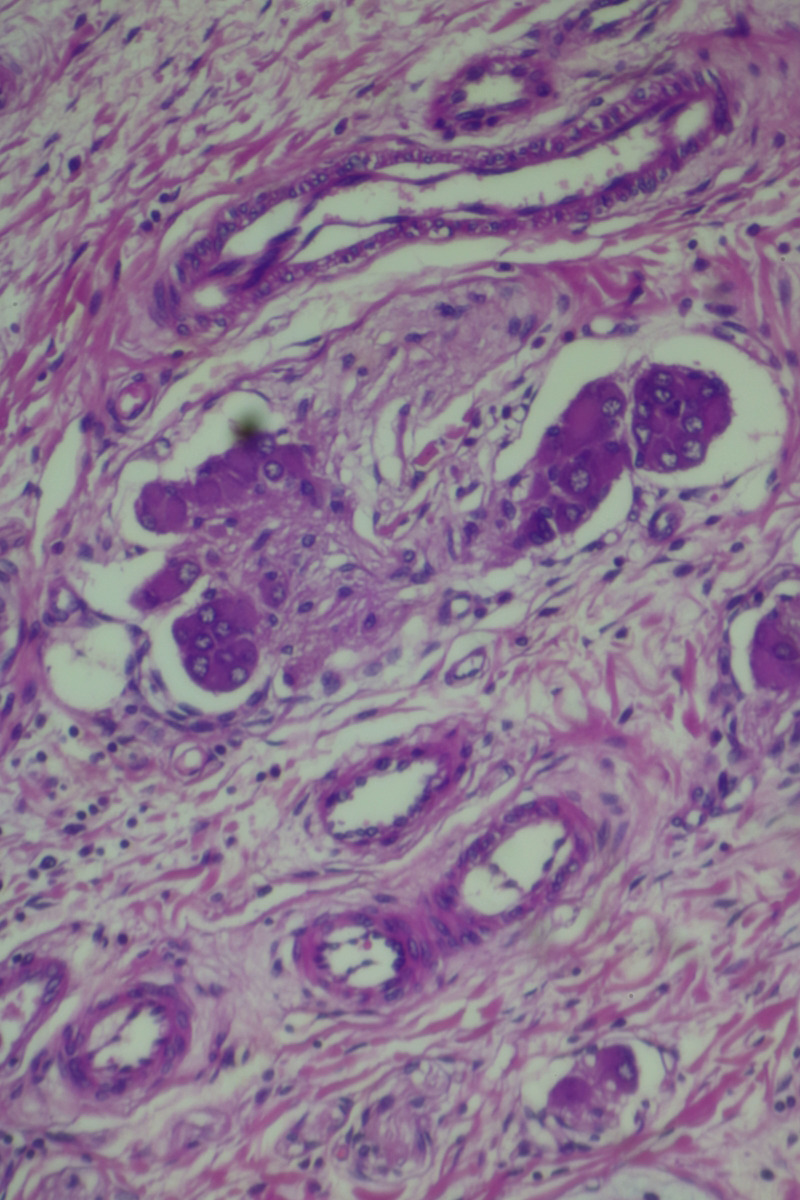
Proliferation of spindle cells, admixed with scattered chronic inflammatory cells throughout the lamina propria. Medium power objective (10×)

**FIGURE 14:**
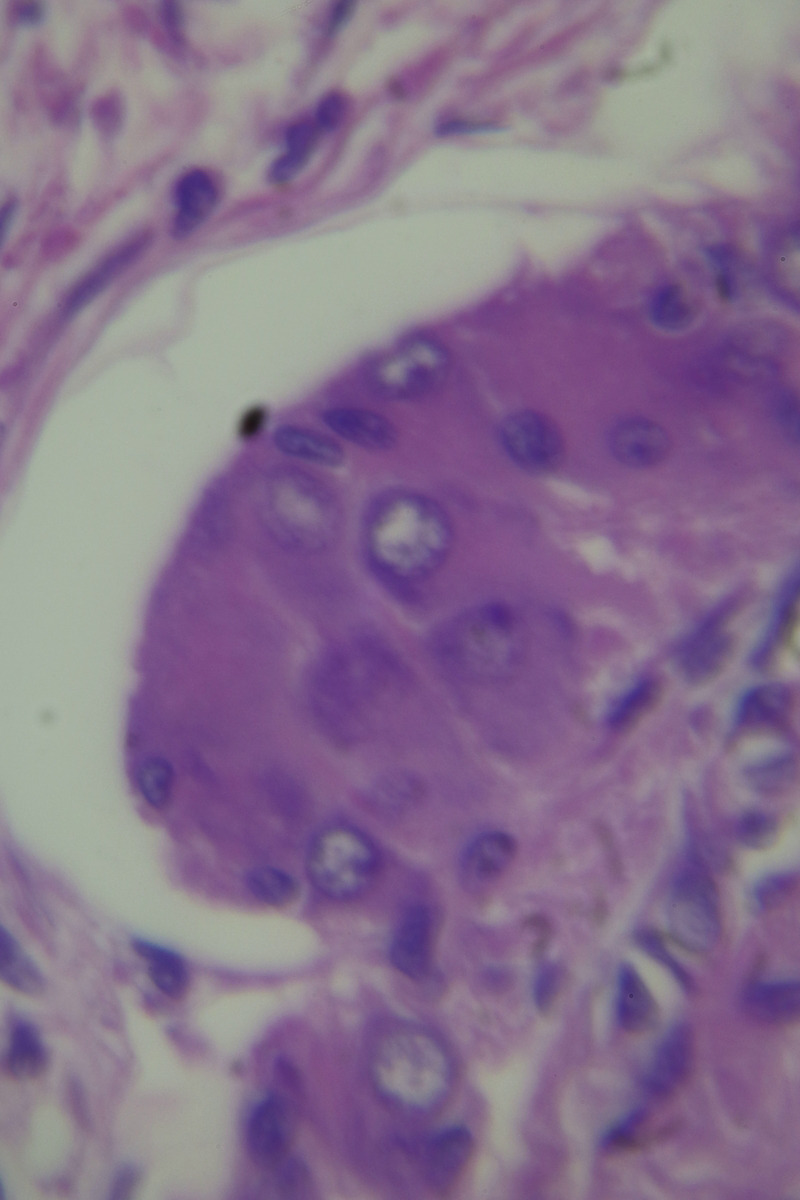
Proliferation of spindle cells, admixed with scattered chronic inflammatory cells throughout the lamina propria. High power objective (40×)

**FIGURE 15:**
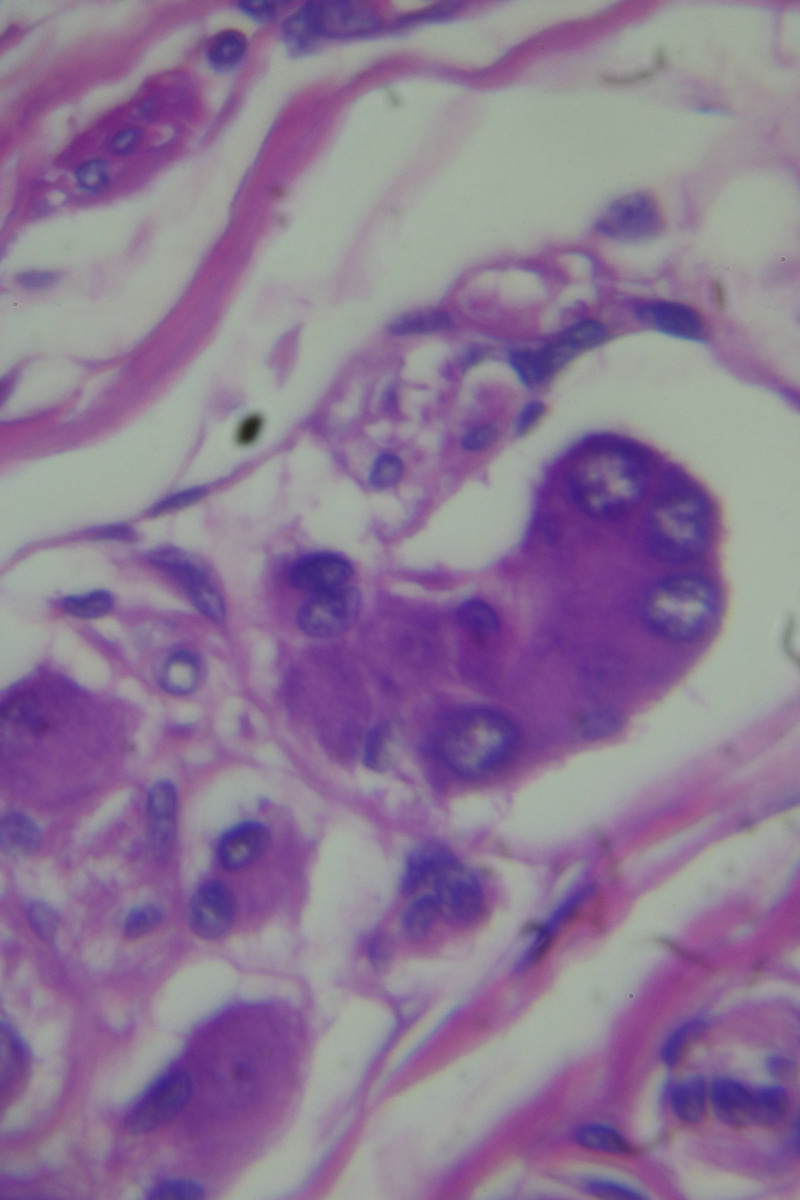
Proliferation of spindle cells, admixed with scattered chronic inflammatory cells throughout the lamina propria. High power objective (40×)

**FIGURE 16:**
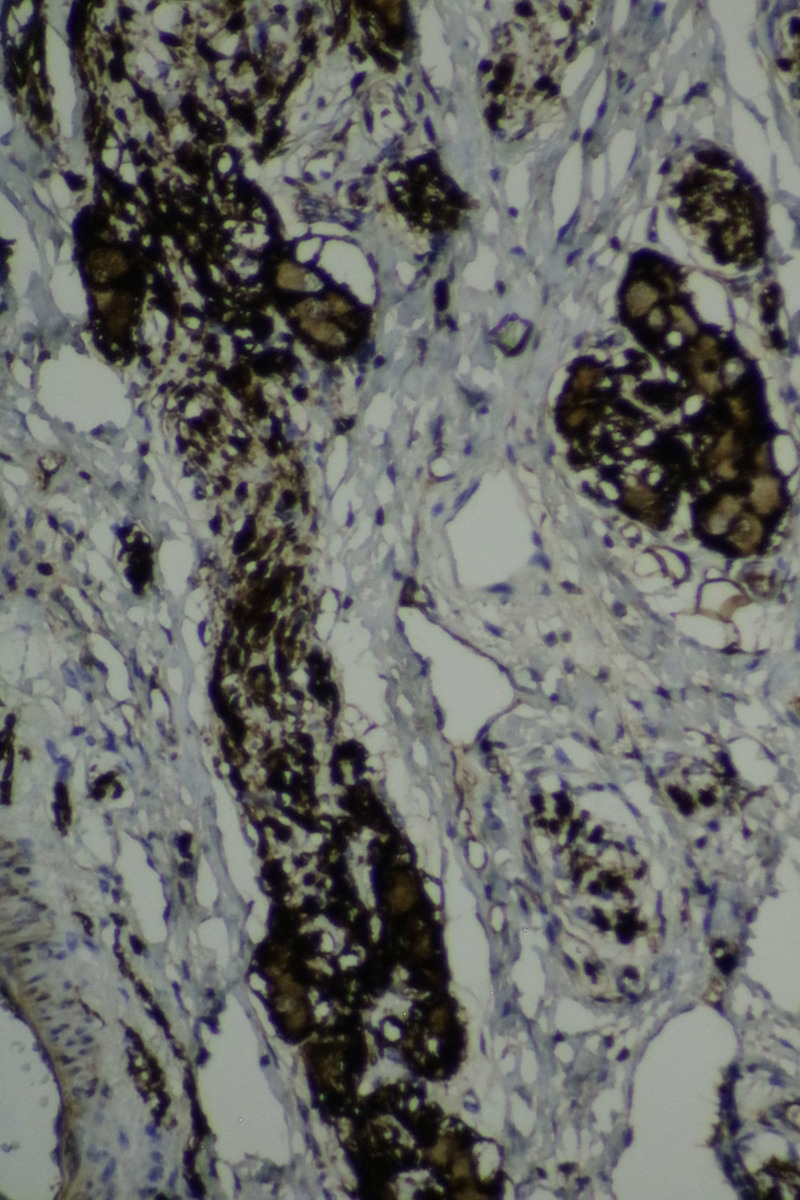
Cells are positive for S‐100 protein immunostaining. Medium power objective (10×)

**FIGURE 17:**
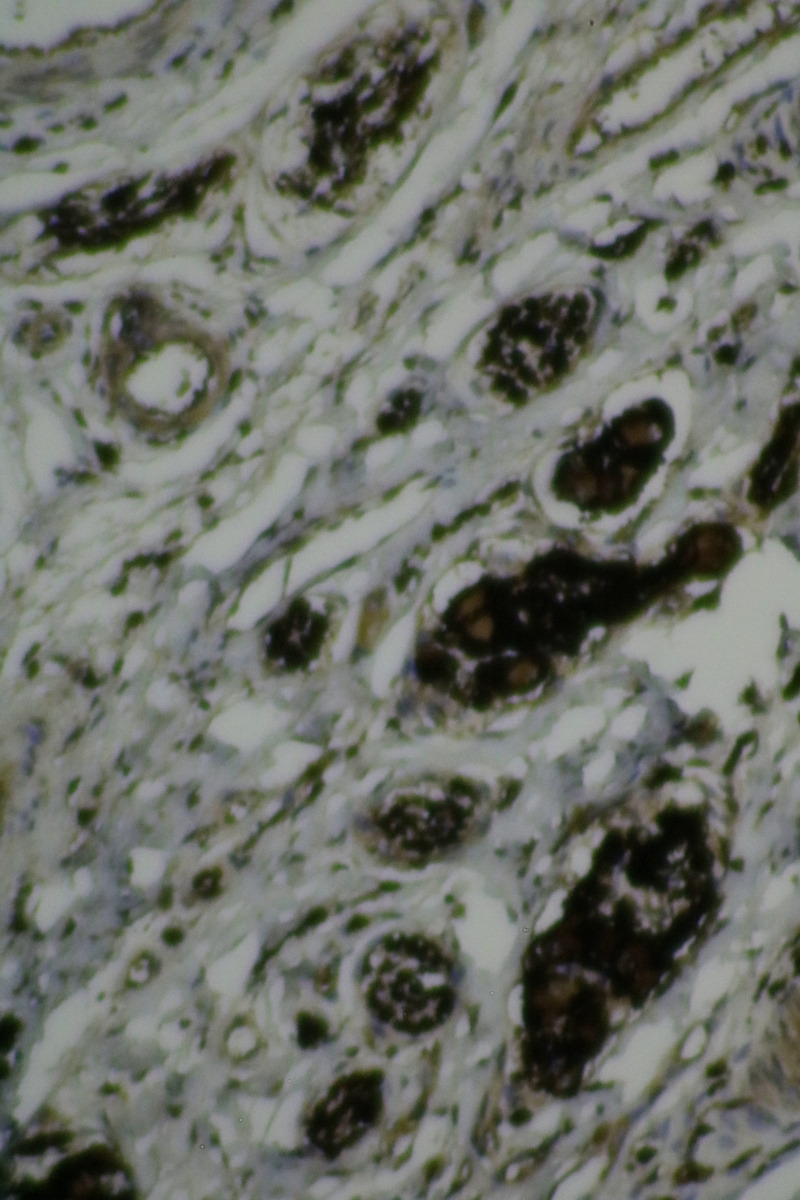
Cells are positive for S‐100 protein immunostaining. Medium power objective (10×)

**FIGURE 18:**
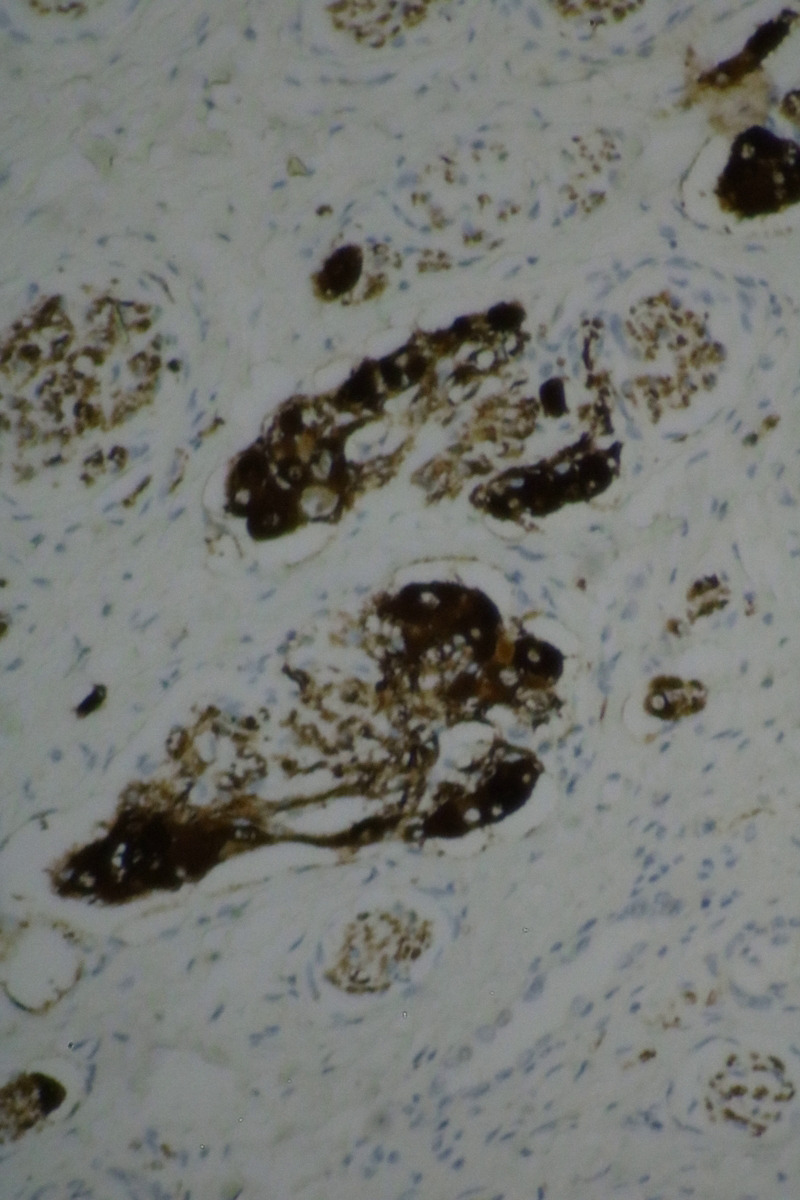
Neurons are positive immunostained for neuron‐specific enolase. Medium power objective (10×)

**FIGURE 19:**
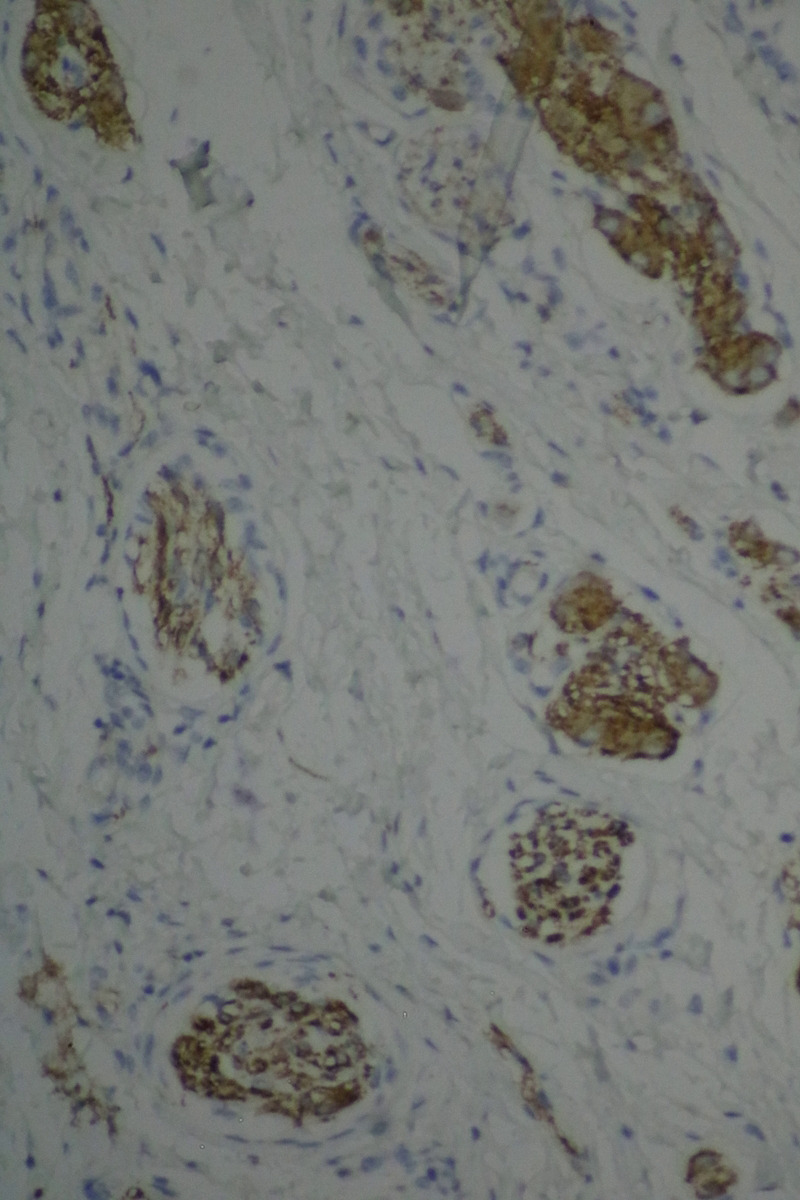
Cells are positive immunostained by synaptophysin. Medium power objective (10×)

The histopathological examination of the specimen revealed macroscopically an area with a double lumen and a thickened wall in the dilated terminal ileum (Figure 8A,B). Microscopically, examination of the sections from the small intestine showed a duplication cyst lined with colonic mucosa. The small intestine, large intestine, and duplication cyst lamina propria were involved with proliferation of spindle cells, admixed with scattered chronic inflammatory cells throughout the lamina propria. The rest of the wall was involved, with a tortuous expansion of multiple variable‐sized nerve fascicles (a plexiform neurofibroma). Reactive lymph nodes were found in the specimen at the mesentery (Figures 9, 10, 11, 12, 13, 14, 15). There was no evidence of malignancy in the examined tissue. The immunohistochemical analysis shows the neural components were positive for S100, CD56, PGP 9.5, synaptophysin, and NSE and focally positive for neurofilaments and negative for chromogranin immunotoxins (Figures 16, 17, 18, 19). The calretinin test confirmed the presence of multiple ganglion cells in the proliferating neural tissue. All are consistent with a diagnosis of diffuse intestinal ganglioneuroma.

The patient was referred to an oncological center for further management; all histopathological slides and specimens have been sent with the patient as well.

## 3 DISCUSSION

Neurofibromatosis is a common autosomal dominant hereditary tumor syndrome, caused by alterations of the neurofibromatosis gene type 1.^4^ They are two types of neurofibromatosis: type 1 and type 2, both of which are autosomal dominant. Ninety‐six percent of neurofibromatosis cases are type 1 (1 in 3,000 births). Neurofibromatosis affects both sexes equally, with no difference in the incidence according to race. Half of the patients with neurofibromatosis have a spontaneous mutation, with the other half due to an inherited gene. Neurofibromatosis type 2 accounts for only 3% of total cases and has a 1 in 33,000 prevalence, with no sex or race association.^5^

Gastrointestinal involvement in neurofibromatosis occurs in three forms: visceral vasculopathy, ganglioneuromatosis, and solitary neurogenic tumors. Intestinal ganglioneuromatosis is a rare condition, which is more common in children than in adults.^6^ Diffuse intestinal ganglioneuromatosis is hamartomatous polyposis that consists of hyperplasia of the enteric nerve fibers and the myenteric plexus. They are well‐differentiated tumors, often associated with multiple endocrine neoplasia and neurofibromatosis.^7^ Ganglioneuromatosis has a very low prevalence.

The clinical presentation of intestinal ganglioneuromatosis is similar to that of intestinal obstructions caused by megacolon, tumors in the ileum or colon, Crohn's disease, tuberculosis, fibrous inflammatory polyps, and multiple schwannomas of the colon. All are differential diagnosis.^8^ Ganglioneuromatosis is differentiated from Crohn's disease and tuberculosis based on histopathology. Ganglioneuromatosis and neurofibromatosis have a wide range of similar clinical features, including constipation, abdominal distension, vomiting, abdominal pain, melena, diarrhea, weight loss, thickening of the bowel wall, and bloody diarrhea.^4^ These wide‐ranging presentations depend on the location and the affected part of the intestine. The condition commonly affects the colon, ileum, appendix, duodenum, and rectum.

In addition to a full laboratory workup and a complete hemogram, the diagnostic workup includes histopathology. It helps the physician to rule out other tumors. A colonoscopy is the most common diagnostic test, although this is difficult among patients of neonatal age. Other radiological investigations include abdominal X‐rays and CT scans.^9^, ^10^ The disease has a high recurrence rate within a short duration of time. Recurrence is determined by the extent of resection done during surgery. For the treatment, extended resection is recommended to involve zones affected microscopically. Postsurgical resection, the condition has a good prognosis, and most patients are discharged within weeks after surgery.

Both ganglioneuromatosis and surgery are associated with a number of significant risks. These include disease recurrence, infection at the surgical site, and the development of intestinal obstructions and volvulus.^9^ Furthermore, ganglioneuromatosis commonly occurs concurrently with other diseases, such as multiple endocrine neoplasias, neurofibromatosis, and colon tumors.^11^ Patients with endocrine neoplasias, neurofibromatosis, and colon tumors are likely to develop ganglioneuromatosis. The clinicopathological correlation between radiology and molecular studies is strongly recommended. Genetic studies and counseling of affected families are also recommended for further screening of other genetic associations and prevention.

## 4 CONCLUSION

The incidence of neonatal intestinal ganglioneuromatosis is very low, and a prompt diagnosis is crucial for its management. Surgical resection is the definitive treatment. The potential association with other diseases complicates the overall management. Neonatal intestinal ganglioneuromatosis and neurofibromatosis require further workups to eliminate other possible associations.

## ETHICAL APPROVAL

Hereby, I, Madani Essa, consciously assure that for the manuscript "Neonatal Intestinal Diffuse Ganglioneuromatosis with Plexiform Neurofibromas; Diagnostic and Management Pitfalls: A Case Report" the following is fulfilled: (1) This material is the authors’ own original work, which has not been previously published elsewhere. (2) The paper is not currently being considered for publication elsewhere. (3) The paper reflects the authors’ own research and analysis in a truthful and complete manner. (4) The results are appropriately placed in the context of prior and existing research. (5) All sources used are properly disclosed (correct citation). Literally copying of text must be indicated as such by using quotation marks and giving proper reference.

## CONSENT

Hereby, I, Madani Essa, confirmed that patient consent has been signed and collected in accordance with the journal's patient consent policy. I confirm that the data supporting the findings of this study are available within the article and its Supplementary material. Raw data that support the findings of this study are available from the corresponding author, upon reasonable request. I confirm that This research received no external funding
